# The COVID-19 Infodemic^*^

**DOI:** 10.26633/RPSP.2021.56

**Published:** 2021-07-06

**Authors:** Sebastián García-Saisó, Myrna Marti, Ian Brooks, Walter H. Curioso, Diego González, Victoria Malek, Felipe Mejía Medina, Carlene Radix, Daniel Otzoy, Soraya Zacarías, Eliane Pereira dos Santos, Marcelo D’Agostino

**Affiliations:** 1 Pan American Health Organization Washington, D.C. United States of America Pan American Health Organization, Washington, D.C., United States of America.; 2 University of Illinois Urbana-Champaign United States of America University of Illinois, Urbana-Champaign, United States of America.; 3 Universidad Continental Lima Peru Universidad Continental, Lima, Peru.; 4 Pan American Health Organization São Paulo Brazil Pan American Health Organization, São Paulo, Brazil.; 5 Organization of Eastern Caribbean States Castries Saint Lucia Organization of Eastern Caribbean States, Castries, Saint Lucia.; 6 Central American Health Informatics Network Guatemala Guatemala Central American Health Informatics Network, Guatemala City, Guatemala.; 7 Ministry of Health of Brazil Brasilia Brazil Ministry of Health of Brazil, Brasilia, Brazil.

On 15 February 2020, during the Munich Security Conference (1), the Director of the World Health Organization (WHO), Dr. Tedros Adhanom Ghebreyesus, stated that the fight against the COVID-19 pandemic was accompanied by a fight against an “infodemic”, leading to a series of initiatives by the WHO and other organizations to face this challenge. This situation is not new: others have occurred during other health emergencies, but never one of the current magnitude, resulting from the increased use of digital applications (2). In the age of digital interdependence, this phenomenon is amplified by the convergence of increased access to mobile devices, internet access, and the use of social networks, which are spreading it like a virus, further and faster than ever before (3).

The term “infodemic” combines the terms “information” and “epidemic” (4) and refers to an excess of information (both true and false) that makes it difficult for people to access reliable sources and obtain valid guidance when it becomes most necessary for decision-making. An infodemic also involves a large increase in the volume of information on a given topic, which can increase exponentially in a very short time when an incident such as the COVID-19 pandemic occurs (3, 4). In this situation, scientific and technical information becomes mixed with rumors, manipulated data, fake expertise, incorrect information, and false and biased news, hindering the recipient’s ability to process and judge it all. Furthermore, access to false or incorrect data produces significant distortions in predictive models, affecting health system planning and decision-making. The scientific community is studying this phenomenon in order to understand its patterns, structures, and characteristics in an attempt to mitigate its consequences and understand people’s behavior when they face it.

The infodemic can negatively impact health and well-being and can also polarize public debate. Campaigns against public health measures, inaccurate or falsified epidemiological data, and fake or biased evidence can potentially alter people’s behavior. This puts extra pressure on health systems, as it undermines the scope and efficiency of the various health intervention programs.

The main factors contributing to the development of the infodemic are associated with a lack of digital literacy programs (5) that address: (a) the difficulties involved in critically searching for, selecting, recommending, and disseminating reliable data and information; (b) a lack of criteria and tools for obtaining critical information in the right format at the right time; and c) poor understanding of the use and relevance of digital applications in health. While these challenges have become an additional burden during the pandemic, they have accelerated the opportunity to educate the population and implement continuing training programs for health workers to develop their skills in the age of digital interdependence (6).

As members of the community, we all have a role in fighting the infodemic, depending on when and where we are dealing with information. This include actions such as: a) determining whether the information makes sense, even if it comes from a reliable source and has been previously shared; (b) confirming the source; (c) participating responsibly in social conversations; and d) above all, when in doubt, choosing not to share information.

As part of the scientific community’s responsibility to deepen our understanding of the infodemic, the Pan American Health Organization, in coordination with WHO, presents this special issue with the most relevant recent studies in the Region of the Americas, providing guidance on a path where there is still much to discover and much to do.
